# Preoperative perforator localization in anterolateral thigh free flap using acoustic doppler and computed tomography angiography

**DOI:** 10.1002/lio2.958

**Published:** 2022-10-26

**Authors:** Sungryeal Kim, Hye Ran Lee, Ju Hyun Yun, Jisun Yang, Jeon Yeob Jang, Yoo Seob Shin, Chul‐Ho Kim

**Affiliations:** ^1^ Department of Otolaryngology, College of Medicine Inha University Incheon South Korea; ^2^ Department of Otorhino‐Laryngology‐Head and Neck Surgery, College of Medicine Catholic Kwandong University Incheon South Korea; ^3^ Department of Otolaryngology, School of Medicine Ajou University Suwon South Korea

**Keywords:** acoustic Doppler sonography, anterolateral thigh free‐flap, CT angiography, head and neck cancer, perforator

## Abstract

**Objectives:**

Our aim in this study was to investigate if we could predict perforator localization during ALTF elevation, using information from acoustic Doppler (AD) and computed tomography angiography (CTA).

**Methods:**

Prospective observational data were collected from H&N cancer patients who received reconstruction with ALTF in Ajou University Hospital Cancer Center from June to December, 2021. Total of 21 cases were included in the analysis. Lower extremity angio‐CT scans were used to determine the course and depth of the perforator before surgery. During intraoperative design of the ALTF, the possible location of the perforator was identified by AD. After flap elevation, the distance between the actual and Doppler‐identified location of the perforator was measured.

**Results:**

The average distance from the actual location to the Doppler‐identified location was 1.29 ± 1.26 cm. Among 21 cases, almost all perforators (20 cases) were identified in a circle with a radius equivalent to the depth of the perforator. Perforator depth measured by CTA showed a significant positive correlation with the distance from the actual to Doppler‐identified location, regardless of skin thickness or body mass index (BMI).

**Conclusions:**

A circle with a radius equivalent to the CTA‐assessed depth of the perforator successfully predicted the location of the perforator in almost all cases. Depth of the perforator measured by CTA combined with Doppler‐identified location can help safely locate the perforator during ALTF harvesting.

Level of Evidence: 4.

## Graphical abstract

The actual location of the perforator in 95% of cases we examined fell within perforator depth distance from Doppler‐identified location. If the information on approximate anatomical variation with CTA such as the perforator depth and real‐time flow information obtained using AD are combined, preoperative prediction of the actual perforator location is possible, preventing damage to the perforator and enabling safer flap elevation.

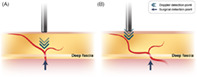

## 1 INTRODUCTION

Since the instruction of the anterolateral thigh free‐flap (ALTF) by Song et al. in 1984,^1^ it has frequently been used for head and neck (H&N) reconstruction due to its numerous advantages. These include (1) a large skin paddle, (2) sufficient volume for reconstruction of H&N defects, (3) a long pedicle, (4) good matching between the branch of the lateral circumflex femoral artery (LCFA) and the caliber of H&N donor vessels such as superior thyroid artery or facial artery, (5) fair chance of primary closure and low donor site morbidity, and (6) ability to use multiple tissue components in various combinations.^2^, ^3^ However, ALTF also has significant drawbacks, with the most severe being, variability in pedicle anatomy. Identifying the location of the perforator can be a serious hurdle for appropriate flap harvesting, especially in patients with a high BMI.

ALTF can be harvested as a subcutaneous flap, a fasciocutaneous flap, or a myocutaneous flap, and the method of finding the perforator varies depending on the desired flap. Two techniques are currently used to find the perforator of ALTF: subfascial and suprafascial approaches. An ALTF acquired through subfascial dissection can provide sufficient volume and deep fascia for large and complex soft tissue defect on oral cavity or pharynx as are present after H&N cancer surgery.^4^ Harvesting of a thin or super‐thin ALTF via suprafascial dissection decreases the donor site morbidities such as deterioration of range of motion, ambulation, and contour aesthetics, but the possibility of partial and/or complete flap loss due to damage to the perforasome higher.^5^, ^6^ Although efforts have been made to understand the perforasome,^7^ an ideal imaging technique for evaluating the perforasome in ALT territory has not yet been identified. Moreover, lipocutaneous shrinkage may occur after adjuvant radiotherapy. It therefore remains challenging to use thin or super‐thin suprafascial dissection for H&N cancer reconstruction surgery.

Furthermore, incidental damage during suprafascial dissection of the subcutaneous tissue for perforator identification can lead to flap failure. Therefore, several tools including acoustic Doppler (AD), color duplex ultrasound, computed tomography (CT), and magnetic resonance (MR) imaging have been used to confirm the location of the perforator.^8^ AD is preferred because of its non‐invasiveness, low costs, small size, and portability.^9^ AD can detect vessels with a diameter of 0.2 mm, with a sensitivity 100% for a skilled operator.^8^, ^10^ However, because of this high sensitivity, it sometimes detects vessels other than the perforator, resulting in a discrepancy between the Doppler‐detected location and the actual perforator as determined by surgical dissection. In addition, it is difficult to track the actual course of the perforator by an AD. CT angiography (CTA) has also been used to assess the anatomical details of blood vessels.^11^ Preoperative CTA can be used to detect atherosclerotic disease, anomaly and the approximate course of the perforator. However, it is difficult to determine the real‐time location of the perforator at the surgical site using CTA. Therefore, there is no current foolproof clinical method for identifying the location of the perforator. In this study, we investigated techniques to determine perforator localization with a focus on using CT‐identified perforator depth and Doppler‐identified location during ALTF elevation.

## 2 MATERIALS AND METHODS

### 2.1 Study participants and data collection

We performed a prospective observational study using data from a total of 21 patients who underwent H&N cancer surgery with ATLF reconstruction from June through December 2021 at Ajou University Hospital. Inclusion criteria were patients who underwent (i) reconstructive surgery with ALTF, and (ii) preoperative lower extremity CTA. There were no specific exclusion criteria. All patients underwent ALTF reconstruction via a subfascial approach for perforator dissection by a single H&N surgeon (Y.S.S.). The study protocol was approved by the Institutional Review Board of Ajou University Hospital (approval no. AJIRB‐MED‐MDB‐21‐629).

### 2.2 Surgical procedure

**FIGURE 1:**
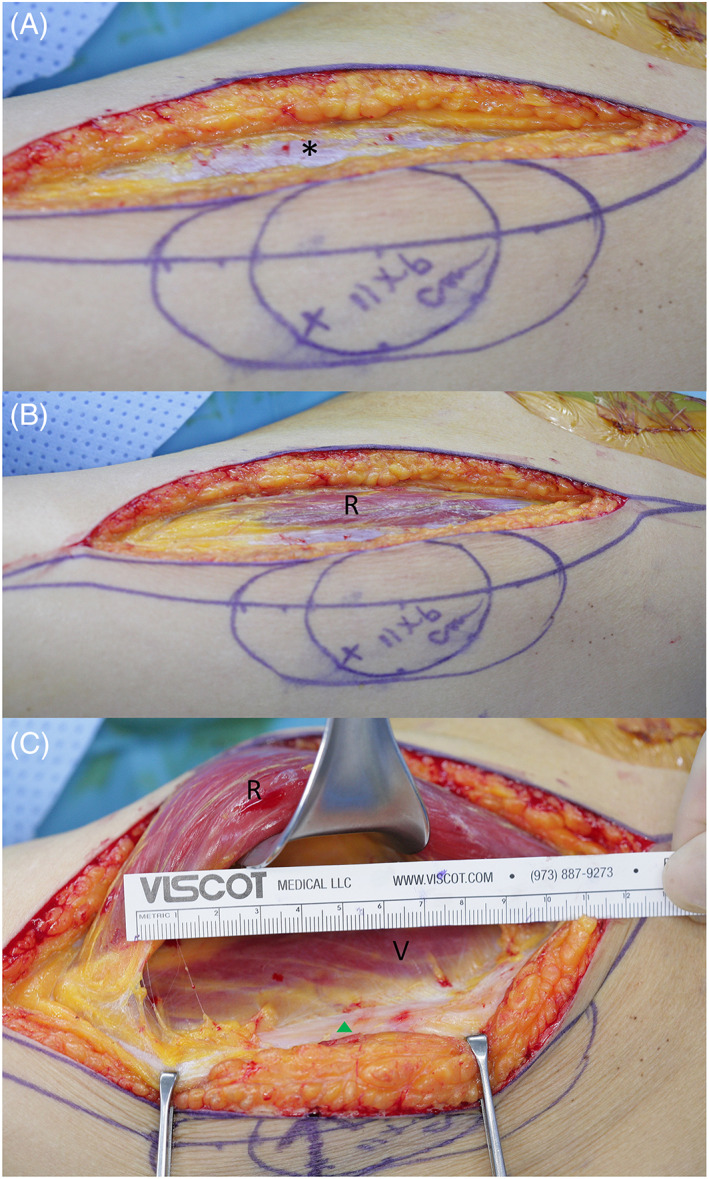
Subfascial dissection technique. (A) After an 11 × 6 cm flap was designed by marking the Doppler‐detected location with “X,” a skin incision in the medial to the anterior superior iliac supine and patella line was performed. *: deep fascia. (B) Incision in the deep fascia for subfascial dissection. (C) Perforator was found (green triangle) by careful subfascial dissection after the rectus femoris was retracted, then the distance between the Doppler‐detected location and actual location was estimated with a ruler. R, rectus femoris; V, vastus lateralis

All ALTFs were harvested by subfascial dissection. Preoperative marking of the ALT perforator was performed using an AD according to the anatomical landmarks described by Wei and colleagues.^12^ After drawing a line from the anterior superior iliac spine and the upper outer border of the patella, a circle with a diameter of 6 cm was drawn based on the midpoint of this line. Within the circle, the perforator location was marked by AD (Minidop ES‐100VX Pocket Doppler with 8 MHz pencil type probe, Hadeco, Inc.). A medial incision was made, then the flap was elevated subfascially (Figure 1). After identification, the rectus femoris was retracted to exposure the intermuscular septum. Careful dissection was performed to find perforators traversing deep fascia. Once the perforator was identified, the distance from the Doppler‐detected location to the actual location of the perforator was measured with a ruler. Skin incisions and the releasing flap were completed and the pedicle was dissected in a retrograde fashion through the descending branch.

### 2.3 Preoperative lower extremity CT angiography evaluation

**FIGURE 2:**
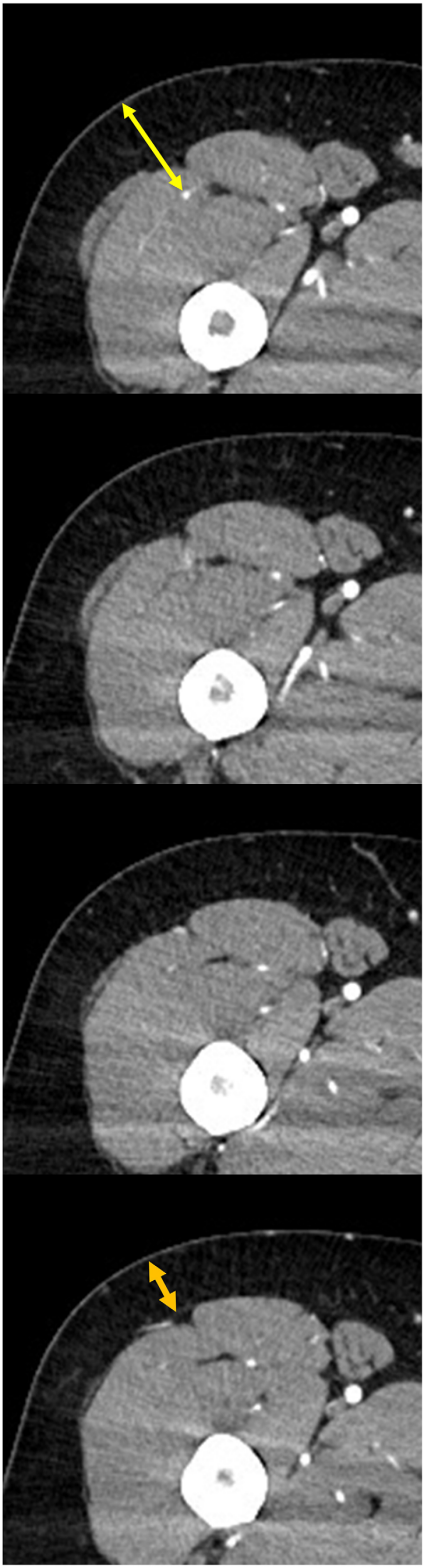
Estimation of skin and perforator depth. Yellow double arrow: perforator depth; orange double arrow: skin depth

All patients underwent preoperative low extremity CTA. CT images were obtained at 0.6 mm collimation and were reconstructed into axial images every 1.5 mm on a 512 × 512 matrix. Arterial CT scans were obtained with 15 s delay after intravenous injection of 130 ml iodinated contrast agent. Venous CT scans with a 120 s delay after arterial scan were obtained. At the point where the perforator was confirmed, the thickness of the skin and depth of the perforator were measured. Skin thickness was measured from the surface of the thigh to the point where the perforator traversed the deep fascia, the perforator depth: from the surface of thigh to the point where the perforator exited out of the descending branch (Figure 2).

## 3 RESULTS

**FIGURE 3:**
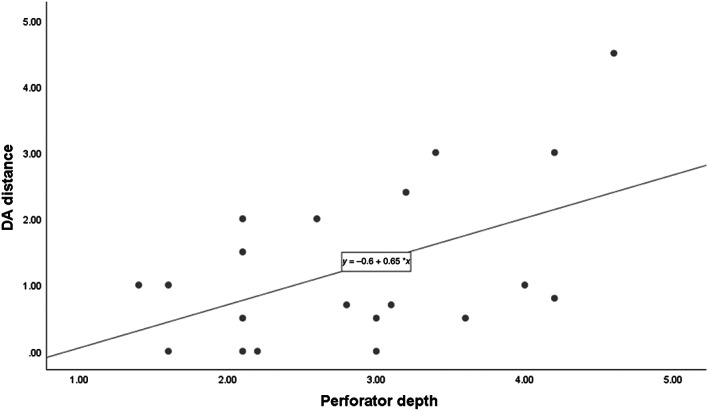
Scatter plot of the distance between Doppler‐detected location and actual location of the perforator. Blue: actual perforator within skin depth distance; red: actual perforator not within skin depth distance but within perforator depth distance

Characteristics of perforators from 21 cases were evaluated (Table 1). Mean patient age was 60 years old and average BMI was 23.49. The actual perforator was observed near the midpoint between the anterior superior iliac spine (ASIS) and patella in 20 of 21 cases. In one case, the actual perforator was not found near the midpoint, but caudal to the midpoint, nearby the ASIS. Thirteen perforators were musculocutaneous type, and seven were septocutaneous type. The average distance between the doppler‐detected location and the actual location of perforator (DA distance) was 1.29 cm. Average thickness of the skin to the deep fascia was 1.07 cm, and the average perforator depth was 2.81 cm. When the relationship between DA distance and perforator/skin depth is evaluated with the Doppler‐detected location as the center of the scatterplot, the actual location of the perforator was within the CTA‐determined perforator depth distance in all cases (Figure 3).

**TABLE 1:** Characteristics of perforators

Sex	Age (years)	Actual perforator	Type of perforator	DA distance (cm)	Skin depth (cm)	Perforator depth (cm)	BMI (kg/m^2^)	Complication
M	36	Yes	Musculocuteneous	1.0	0.6	1.6	28.2	No
M	80	Yes	Septocutaneous	2.0	0.8	2.1	23.9	No
M	65	Yes	Septocutaneous	0.0	0.9	3.0	15.5	No
M	61	Yes	Musculocuteneous	2.4	0.8	3.2	28.6	No
M	80	Yes	Septocutaneous	1.0	0.5	4.0	23.0	No
M	60	No	N/A	N/A	0.6	2.9	21.8	No
M	74	Yes	Septocutaneous	0.0	1.4	2.1	28.9	No
F	61	Yes	Musculocuteneous	0.8	2.8	4.2	28.2	No
M	49	Yes	Musculocuteneous	4.5	1.1	4.6	27.2	No
F	59	Yes	Musculocuteneous	0.0	1.1	2.2	20.9	No
F	49	Yes	Musculocuteneous	0.5	1.7	2.1	23.1	No
F	62	Yes	Musculocuteneous	0.0	0.9	1.6	18.9	No
M	80	Yes	Septocutaneous	1.0	0.5	1.4	22.8	No
M	43	Yes	Musculocuteneous	0.7	0.8	3.1	23.4	No
M	75	Yes	Musculocuteneous	3.0	0.7	4.2	21.0	No
M	55	Yes	Musculocuteneous	3.0	0.4	3.4	21.4	No
M	56	Yes	Musculocuteneous	0.5	0.6	3.0	26.5	No
F	59	Yes	Septocutaneous	2.0	1.6	2.6	25.0	No
M	58	Yes	Musculocuteneous	1.5	0.3	2.1	19.0	No
F	58	Yes	Septocutaneous	0.5	2.4	3.6	25.7	No
F	62	No	Musculocuteneous	0.7	1.5	2.8	20.4	No
Mean ± standard deviation (cm)				1.29 ± 1.26	1.07 ± 0.67	2.81 ± 0.99	23.49 ± 3.67	

Abbreviations: BMI, body mass index; DA, from doppler‐detected point to actual perforator.

**FIGURE 4:**
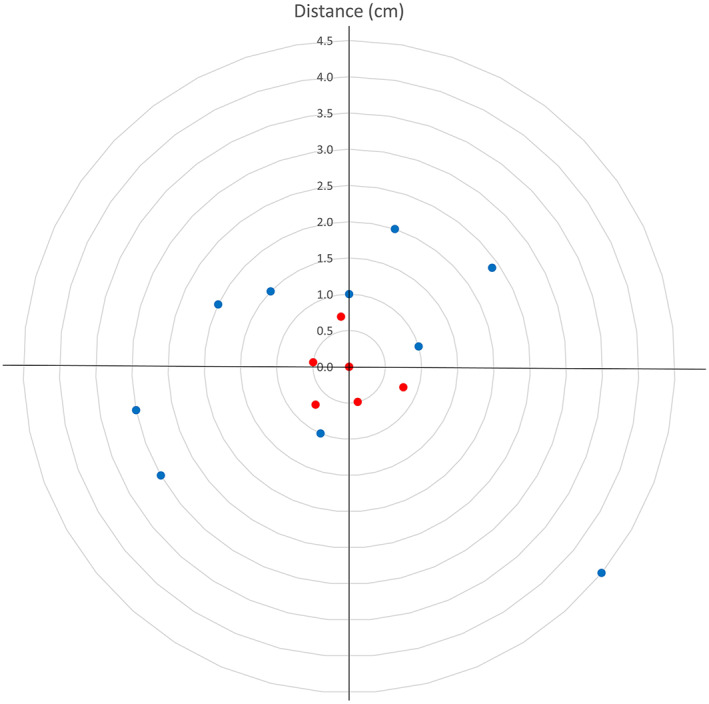
Correlation between the Doppler‐detected location and actual location of the perforator and perforator depth

Correlation analysis was conducted to determine how skin depth, perforator depth, and BMI affected DA distance (Table 2). Skin depth and BMI were not related to DA distance, whereas perforator depth showed a significant positive correlation with DA distance. In addition, a regression analysis was performed between DA distance and perforator depth, and the *p* value was .022 (Figure 4).

**TABLE 2:** Correlations between the distance from the Doppler‐detected location to the actual location of the perforator, perforator/skin depth, and BMI

	Pearson's coefficient	*p*‐Value
Skin depth	−0.238	.312
Perforator depth	0.509	.022
BMI	0.204	.388

Abbreviation: BMI, body mass index.

## 4 DISCUSSION

Two approaches are currently used to find the perforator for ALTF: subfascial and suprafascial approaches. Suprafascial dissection can provide a thin, pliable flap and is associated with fewer donor site morbidities such as compartment syndrome, muscle herniation/bulging or abnormal sensations in the lower extremity, even though a skin graft might be required.^13^, ^14^ However, a major surgical risk associated with the suprafascial approach is related to the perforasome, a unique vascular territory supplied by a single perforator.^15^ Schaverien et al. classified perforasomes into three types.^7^ In type 3 perforasomes, the perforator is divided into several branches at the suprafascial level, and can be easily damaged by suprafascial dissection, resulting in marginal necrosis of the flap.^16^ In reconstruction after H&N cancer surgery, marginal necrosis can lead to a fistula, which adversely affects clinical outcomes. According to Chen et al., fistulas were more frequent in patients who had undergone suprafascial dissection than those who had undergone subfascial dissection (10% of cases versus 6.5%, respectively), suggesting that suprafascial dissection in H&N reconstruction may be less safe than subfascial dissection.^13^ A subfascial approach can preserve the perforasome, and is often simpler than suprafascial dissection decreasing operation time. In addition, the deep fascia obtained with subfascial dissection may be useful in the reconstruction of H&N defects. A waterproof suture line can be made with deep fascia, and in cases of an unstable hyoid due to suprahyoid muscle resection, the deep fascia can also be used for hyoid suspension.^3^

One of the main hurdles in ALTF is anatomical variation of the perforator, which cannot be predicted preoperatively. In a systematic review conducted by Smith et al., the overall mean number of perforators was 2.3, but no perforator was found in 2% of patients.^17^ Yu et al. investigated the presence or absence of a perforator in 100 patients from a Western population according to an ABC system they devised (Perforator B: near the midpoint of the line from the anterior superior iliac spine to the upper lateral border of the patella, Perforator A and C: 5 cm proximal and distal to perforator B). Perforator B was not found in 11 of 100 patients, but perforators A or C were present in those cases.^18^ Due to the anatomic variability of perforator, efforts have been made to determine the exact location of the perforator before surgery using various tools such as AD, color Doppler, catheter angiography, and CTA. Cheng et al. reported that the sensitivity, specificity, and accuracy of AD was 89.7%, 18.2%, and 80.5%, respectively.^19^ Because color Doppler can directly check vascular flow, the sensitivity is reported to be higher than that of AD, but scanning skill is required.^20^ CTA had a sensitivity of 90.4% in a meta‐analysis.^21^ However, it is not conducted in real‐time and has disadvantages such as contrast medium allergy, potential carcinogenic effects due to radiation exposure, and its time consuming nature.^11^ Catheter angiography using indocyanine, indocyanine green angiography, also showed perforator localization of nearly 100%, and it has the advantage of perforator mapping and perfusion in real time, but it is expensive and invasive.^11^, ^22^ Each test has advantages and disadvantages, so one or two tests are usually performed considering situations such as patients, preference of surgeon, and environment of operating room.

**FIGURE 5:**
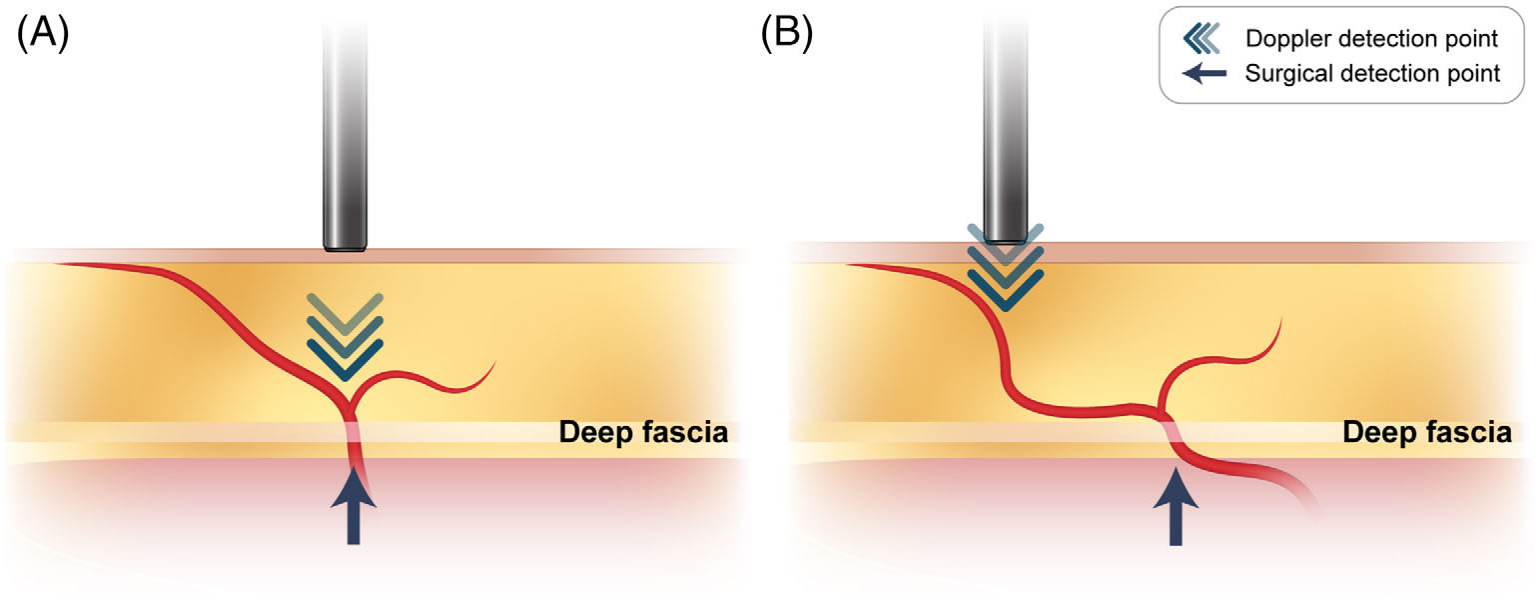
Schematic illustration of points that can be detected by acoustic Doppler

Among the methods used to evaluate the location of the perforator before surgery, AD is simple to use and provides relatively easy‐to‐interpret results, but can detect non‐usable blood vessels and only flow can only be assessed, making it difficult to trace the actual perforator.^23^ Moreover, due to Doppler's principle, the Doppler‐detected location may differ from the actual perforator location. AD uses the principle that the frequency changes when ultrasonic waves collide with a moving object and are reflected. This changed frequency is called the “shifted frequency” and is affected by the speed of the moving object and the angle between the emitted ultrasonic wave and the moving object. As the angle approaches 90°, the shifted frequency decreases, and as the angle approaches 0°, the shifted frequency increases. Therefore, in the case of a blood vessel parallel to the transducer, the sound produced by AD increases. Blood vessels parallel to the transducer include the perforator and communicating branches that connect to the subdermal plexus as well as the perforator (Figure 5). In addition, blood vessels in the muscle can also be detected because the 8 MHz transducer usually used in ALTF has a penetration depth of 3.5 cm.^7^, ^24^ However, if the perforator is located deep within the tissue, the course of blood vessel can be meandering.^11^ To reduce the sensitivity of AD to deep blood vessels, Mun et al. suggested that it is possible to distinguish whether a perforator is a perforator or not by the change in sound when the transducer is compressed.^25^ CTA enables three‐dimensional visualization of the perforator, so information such as vascular variation and course can be obtained.^8^ It takes less than 10 min to check the anatomical information of blood vessels and measure the depth of the perforator with CTA, and it does not delay the operation because it can be measured before the operation starts. However, there is a disadvantage that an additional cost must be paid for the CT scan.

We demonstrated that in 95% of cases we evaluated, the actual perforator was within the perforator depth distance from Doppler‐detected location. These findings indicate that AD and CTA can be used as complementary tools to identify perforator location.

In the current investigation, among 21 cases, 20 perforators (95%) were identified within a circle with a radius equivalent to the depth of the perforator and center at doppler detected point in 20 cases (95%). Perforator depth measured by CTA showed a significant positive correlation with distance from the actual to the Doppler‐identified location, regardless of skin thickness or BMI. BMI affects perforator prediction with AD. Yu et al. reported that the probability of correct detection of perforator location decreased as the BMI increased. In patients with thick subcutaneous tissue, the skin perforator would appear more oblique than in thin patients, resulting in a discrepancy between the Doppler‐detected location and the actual perforator location.^26^ However, the accuracy of the AD is less than 100% even in Asians with a lower BMI than Western populations.^27^, ^28^ Shaw et al. reported that pure muscular perforators or the main descending branch caused false positives in lean patients, resulting in an increase in false‐positive or false‐negative findings in lean or obese patients and fewer false‐positive or ‐negative results in those individuals with a normal body habitus.^29^ Considering the penetration depth of AD, blood vessels under the deep fascia can be detected in thin‐skinned patients. Here we demonstrated that discrepancies between the Doppler‐detected location and actual perforator location were related to perforator depth, not skin depth or BMI. When considering the course of the perforator, both the subcutaneous fat and muscle mass of the thigh affect the perforator depth. Therefore, it will be difficult to predict the perforator depth with the skin depth which reflects only the subcutaneous fat and BMI that is not specific to thigh. Although, CTA can accurately measure the depth of the perforator, it would be good if there is an alternative tool considering the cost and consuming time of CTA. Thigh circumference is utilized to measure total or regional muscle mass in anthropometry.^30^ It is specific to the thigh, and contains information on both muscle and subcutaneous fat. Therefore, it will be worth to study the effectiveness of thigh circumference in predicting perforator depth.

Even if the existence of a perforator has been sufficiently evaluated preoperative, there may be no perforator near the midpoint of thigh. Surgeons should proceed with the operation keeping in mind that there may not be a perforator, even if there is preoperative evidence that a perforator is present.

This study had several limitations. First, this study is a retrospective study with a small sample size at a single institution. Second, factors such as operation time were not included in this study. This study is a preliminary study, with a small number of cases, but it was conducted on a scale enough to show statistical significance.^31^ In this study, it was concluded that the depth and location of the perforator were significant, so in future studies, more cases can be analyzed including variables such as operation time. Third, all patients were Asian. Since the thickness of the anterolateral thigh is known to be different between Asians and Westerners, if the relationship between the depth and position of the perforator is studied in Westerners, the perforator can be found more safely.

## 5 CONCLUSION

We demonstrated that CTA and AD can be used as complementary tools to identify perforator location. The actual location of the perforator in 95% of cases we examined fell within perforator depth distance from Doppler‐identified location. The perforator depth on CTA combined with the AD can help surgeons safely locate the perforator during ALTF harvesting. The error between the predicted location and the actual location of the perforator was confirmed through CTA in this study, but in further studies, it would be beneficial to both the surgeon and the patient if the error could be confirmed with a simpler and more intuitive test such as a physical examination.

## CONFLICT OF INTEREST

The authors declare that they have no known competing financial interests or personal relationships that could have appeared to influence the work reported in this article.
